# Supplementary figures and images for: ATG7/GAPLINC/IRF3 axis plays a critical role in regulating pathogenesis of influenza A virus
Source: PLoS Pathog. 2024 Jan 16;20(1):e1011958. doi: 10.1371/journal.ppat.1011958 (PMC10817227; doi:10.1371/journal.ppat.1011958)

# S1 Fig

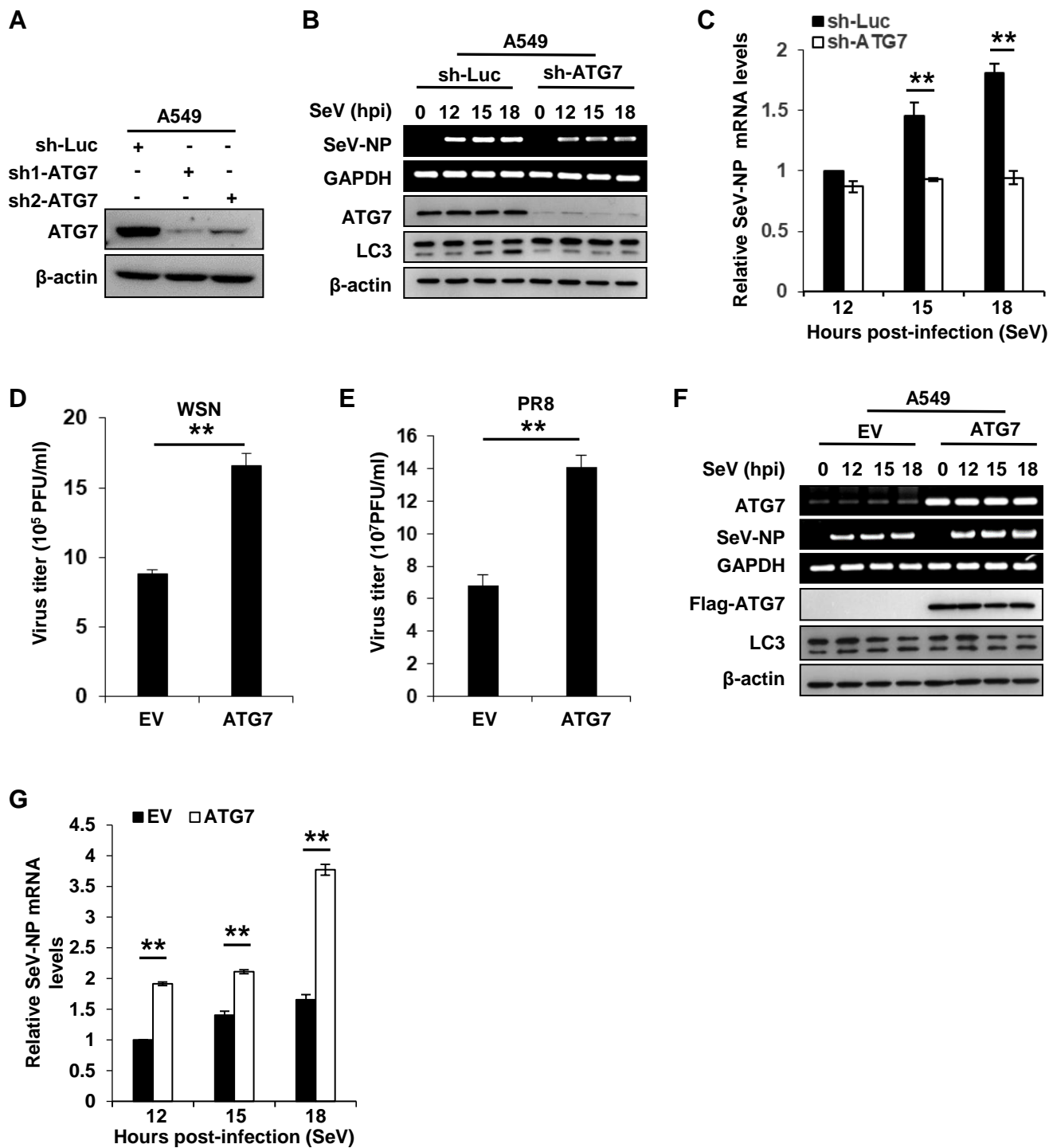

Supplement: S1 Fig — (A) ATG7 protein levels in A549 cells stably expressing control or ATG7 shRNAs were examined by Western blotting. (B and C) Control and ATG7 knockdown A549 cells were infected with SeV (MOI = 0.5) for the indicated time. Viral NP RNA levels in the cells were examined by RT-PCR (B) and qRT-PCR (C) respectively. (D and E) Control and ATG7 overexpressing A549 cells were infected with WSN (MOI = 0.5) (D), or PR8 (MOI = 0.2) for 16 h (E). The supernatants were collected for plaque forming assay. (F and G) Control and ATG7 overexpressing A549 cells were infected with SeV (MOI = 0.5) for the indicated time. RT-PCR (F) and qRT-PCR (G) were performed to test viral NP RNA levels in the cells. Data are presented as means ± SD from three independent experiments, **p < 0.01. (PDF) [file ppat.1011958.s001.pdf]

S2 Fig

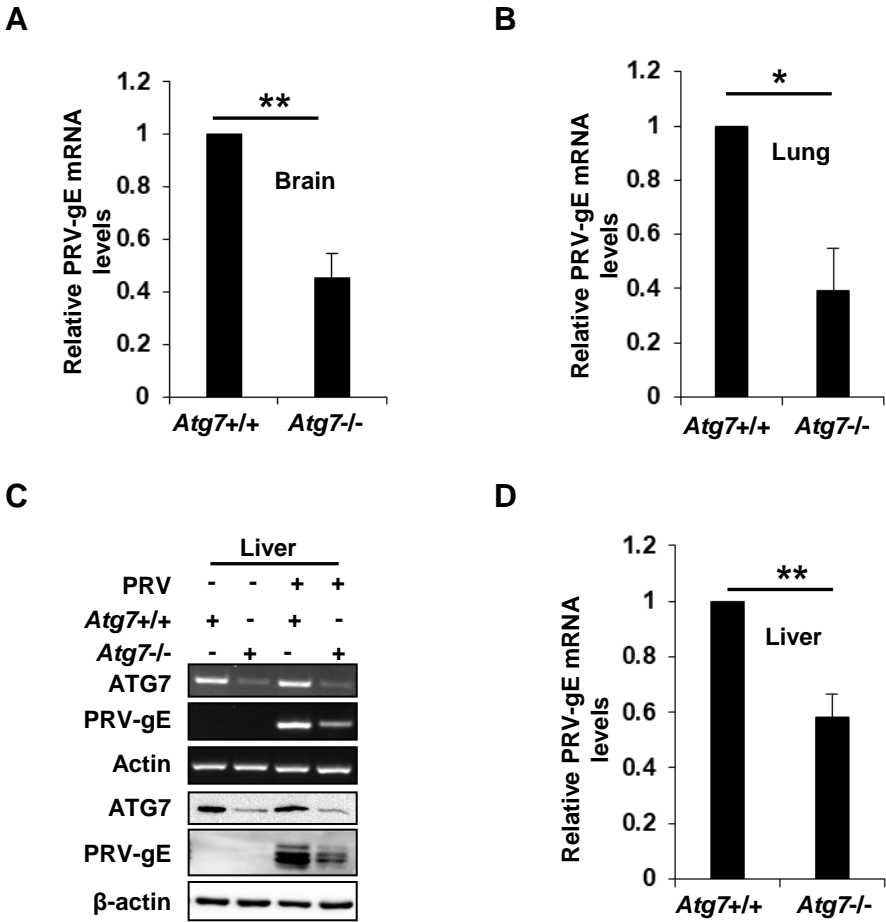

Supplement: S2 Fig — (A-D) WT and ATG7 CKO mice were injected intramuscularly with 1×10^6^ PFU of PRV. Viral gE RNA levels in the brains (A) and lungs (B) from WT and ATG7 CKO mice at 2 dpi were tested by qRT-PCR. Viral gE protein levels in the livers from WT and ATG7 CKO mice at 2 dpi were determined by Western blotting (C), and gE RNA levels were tested by RT-PCR (C) and qRT-PCR (D) respectively. RT-PCR and Western blotting data were repeated independently three times with similar results. Shown are representative data of three biologically independent experiments. Data are presented as means ± SD from three independent experiments, *p < 0.05,**p < 0.01. (PDF) [file ppat.1011958.s002.pdf]

S3 Fig

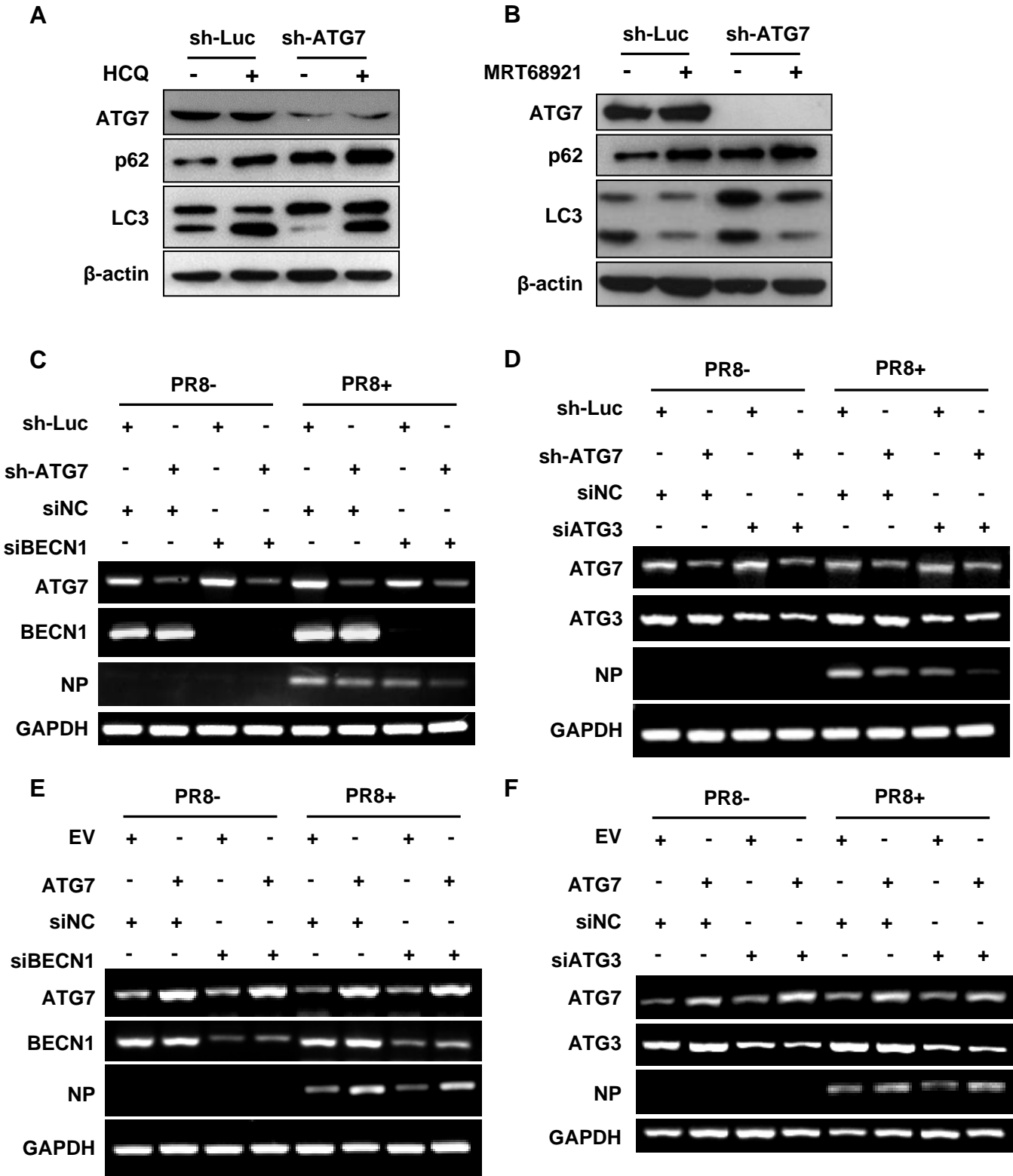

Supplement: S3 Fig — (A and B) Control and ATG7 knockdown A549 cells were pretreated with HCQ (20 μM) (A) or MRT68921 (5 μM) (B) for 3 h, and then infected with PR8 (MOI = 0.2) for 16 h. The levels of p62 and LC3 in the cells were detected by Western blotting. (C and D) Control and ATG7 knockdown A549 cells were transfected with siRNA targeting BECN1 (C) or ATG3 (D), followed by infection with PR8 (MOI = 0.2) for 16 h. The RNA levels of viral NP were detected by RT-PCR. (E and F) Control and ATG7 overexpressing A549 cells were transfected with siRNA targeting BECN1 (E) or ATG3 (F), followed by infection with PR8 (MOI = 0.2) for 16 h. The RNA levels of viral NP were detected by RT-PCR. RT-PCR and Western blotting data were repeated independently three times with similar results. Shown are representative data of three biologically independent experiments. (PDF) [file ppat.1011958.s003.pdf]

# S4 Fig

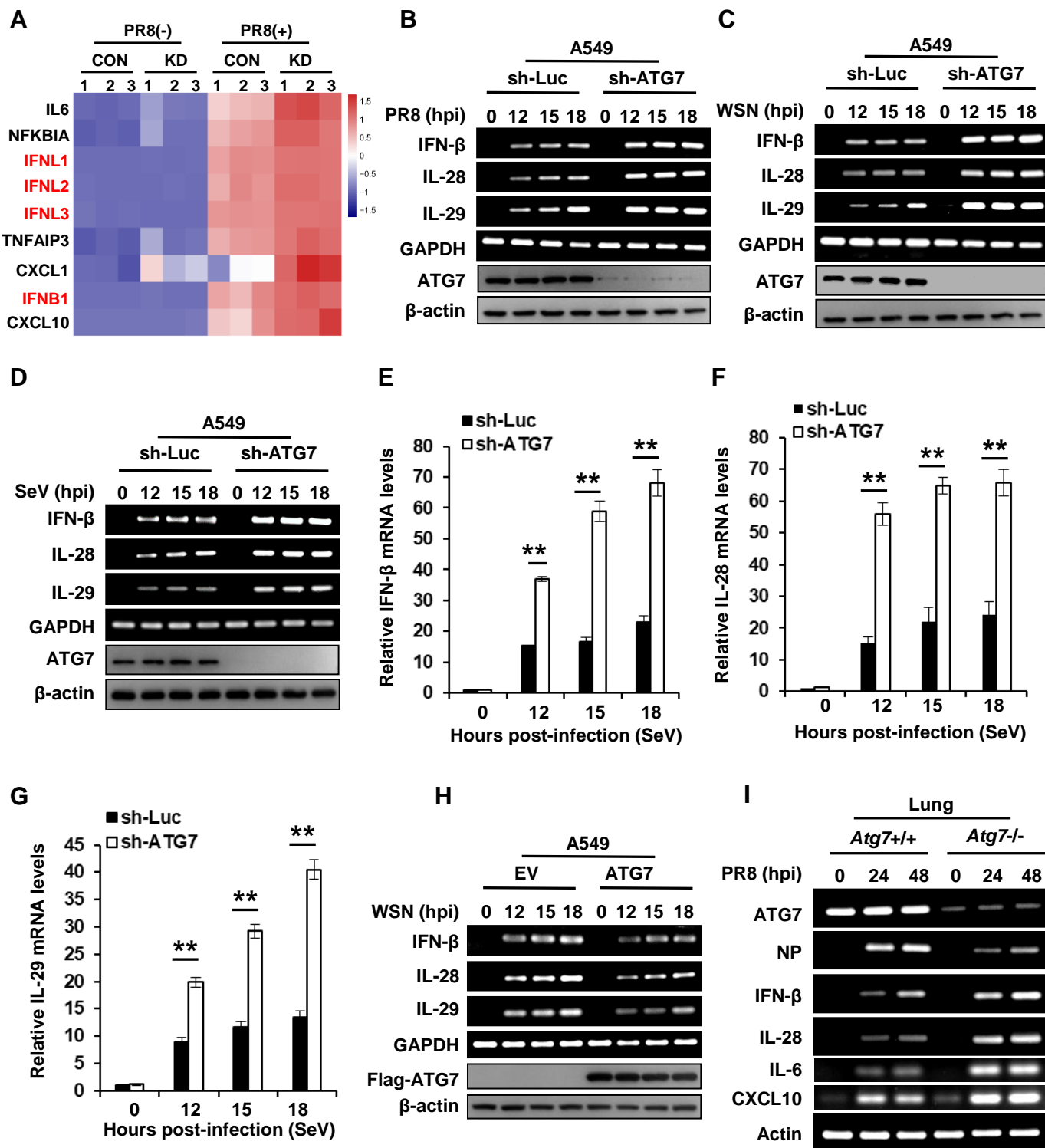

Supplement: S4 Fig — (A) Transcriptome RNA sequencing analysis of control and ATG7 knockdown A549 cells infected with or without PR8 (MOI = 0.5) for 16 h. (B and C) Control and ATG7 knockdown A549 cells were infected with PR8 (MOI = 0.2) (B), or WSN (MOI = 0.5) (C) for the indicated time. The RNA levels of IFN-β, IL-28 and IL-29 were examined by RT-PCR. (D-G) Control and ATG7 knockdown A549 cells were infected with SeV (MOI = 0.5) for the indicated time. The RNA levels of IFN-β, IL-28 and IL-29 were examined by RT-PCR (D) and qRT-PCR (E-G) respectively. (H) Control and ATG7 overexpressing A549 cells were infected with WSN (MOI = 0.5) for the indicated time. The RNA levels of IFN-β, IL-28 and IL-29 were examined by RT-PCR. (I) WT and ATG7 CKO mice were infected with PR8 (5×10^4^ PFU) for 0, 24, and 48 h. The RNA levels of IFN-β, IL-28, IL-6 and CXCL10 in the lungs of mice were examined by RT-PCR. RT-PCR and Western blotting data were repeated independently three times with similar results. Shown are representative data of three biologically independent experiments. Data are presented as means ± SD from three independent experiments, **p < 0.01. (PDF) [file ppat.1011958.s004.pdf]

S5 Fig

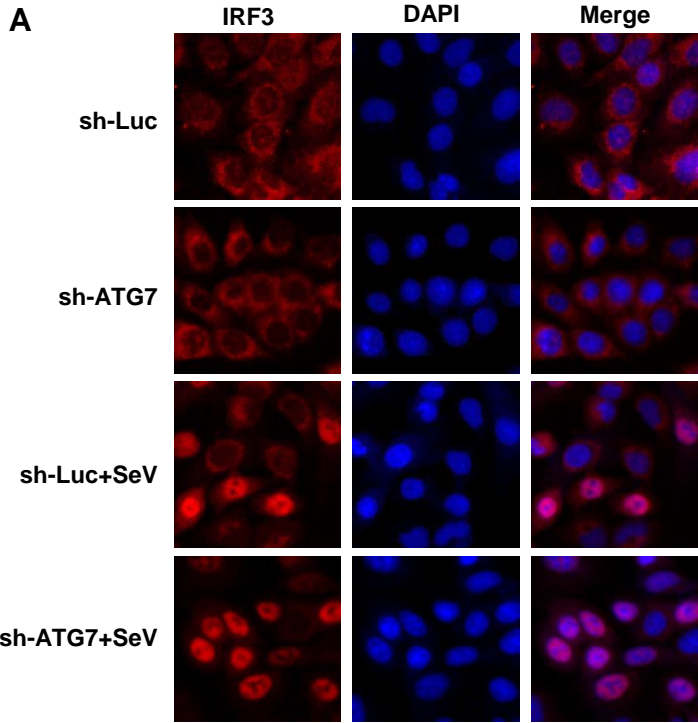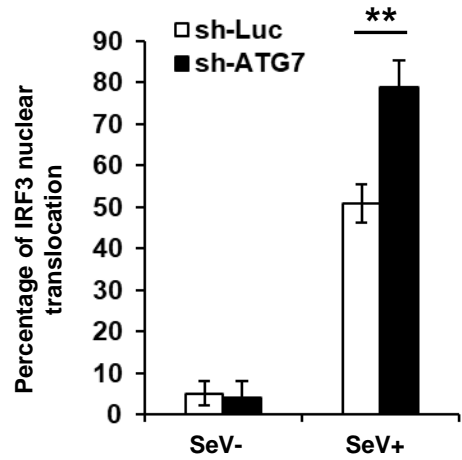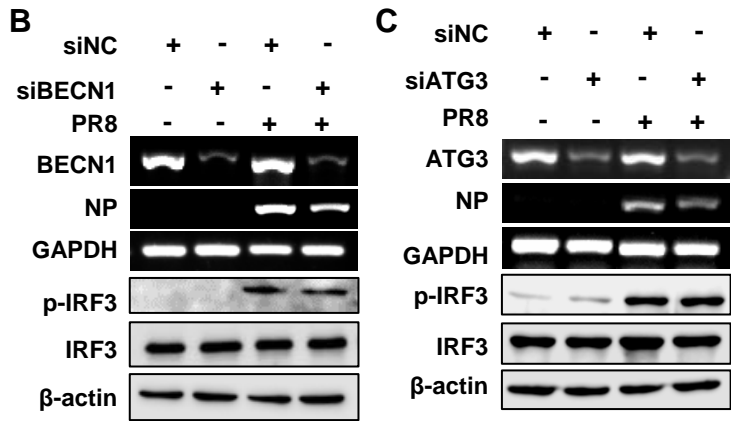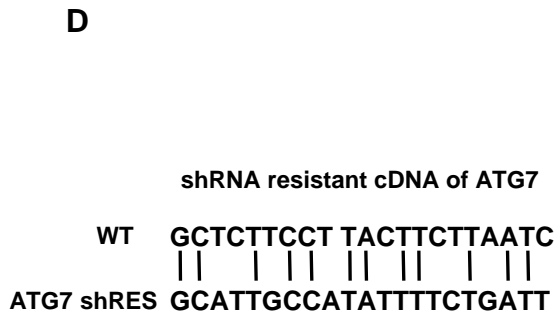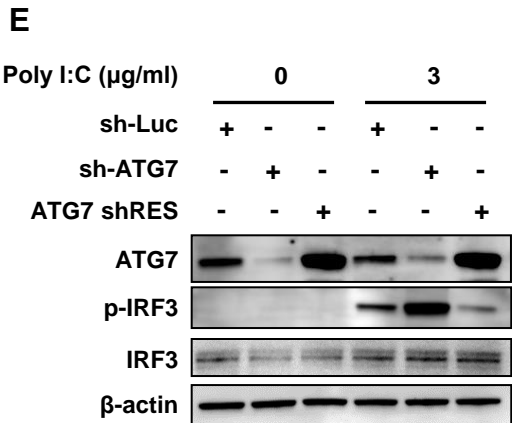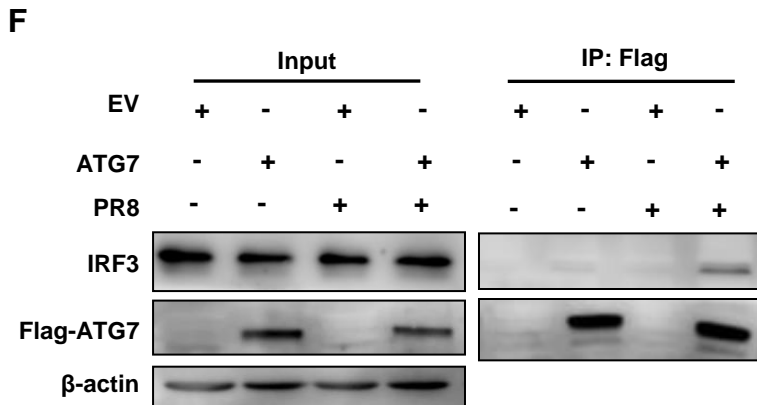

Supplement: S5 Fig — (A) Control and ATG7 knockdown A549 cells were infected with SeV for 12 h, and then subjected to immunofluorescence assays with anti-IRF3 antibody (left). Nuclei were visualized with DAPI (blue). Shown are percentages of cells with IRF3 located in the nuclei (right). Data are presented as means ± SD, **p < 0.01. (B) A549 cells were transfected with control siRNA or BECN1 siRNA, followed by infection with PR8 (MOI = 0.2) for 16 h. The phosphorylated IRF3 (p-IRF3) levels in the cells were examined by Western blotting. (C) A549 cells were transfected with control siRNA or ATG3 siRNA, followed by infection with PR8 (MOI = 0.2) for 16 h, and the p-IRF3 levels in the cells were examined by Western blotting. (D-E) Control and ATG7 knockdown A549 cells re-expressing an shRNA-resistant form of ATG7, were transfected with poly(I:C) for 4 h. The levels of p-IRF3 in the cells were examined by Western blotting. (F) Control and ATG7 overexpressing A549 cells were infected with PR8 (MOI = 0.5) for 12 h, followed by immunoprecipitation (IP) assays using Flag antibody. IRF3 and Flag-ATG7 levels were examined by Western blotting. (PDF) [file ppat.1011958.s005.pdf]

# S6 Fig

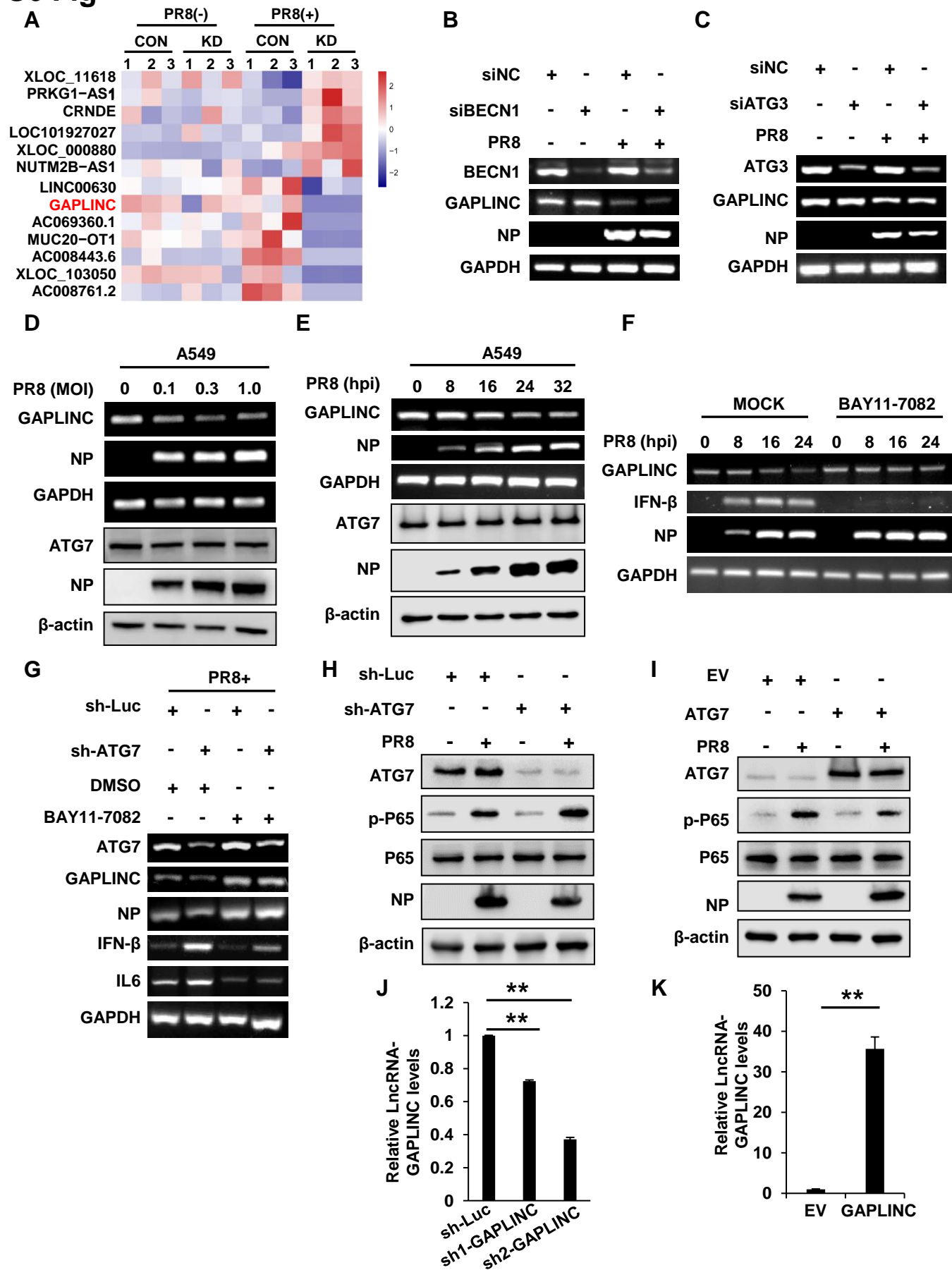

Supplement: S6 Fig — (A) Transcriptome RNA sequencing analysis of lncRNAs expression in control and ATG7 knockdown A549 cells infected with or without PR8 (MOI = 0.5) for 16 h. (B and C) A549 cells were transfected with BECN1 siRNA (B) or ATG3 siRNA (C), followed by infection with PR8 (MOI = 0.2) for 16 h. GAPLINC RNA levels in the cells were examined by RT-PCR. (D and E) A549 cells were infected with PR8 at indicated MOIs for 16 h (D), or at an MOI of 0.2 for the indicated time (E). RT-PCR and Western blotting were performed to examine GAPLINC RNA levels and ATG7 protein levels respectively. (F) A549 cells were pretreated with BAY-117082 (50 nmol/ml) or DMSO for 1 h, followed by infection with PR8 (MOI = 0.2) for the indicated time. The RNA levels of GAPLINC were examined by RT-PCR. (G) Control and ATG7 knockdown A549 cells were pretreated with BAY-117082 (50 nmol/ml) or DMSO for 1 h, followed by infection with PR8 (MOI = 0.2) for 16 h. The GAPLINC RNA levels were detected by RT-PCR. (H) Control and ATG7 knockdown A549 cells were infected with PR8 (MOI = 0.2) for 16 h. The levels of P65 phosphorylation (p-P65) in the cells were examined by Western blotting. (I) Control and ATG7 overexpressing A549 cells were infected with PR8 (MOI = 0.2) for 16 h. The levels of p-P65 in the cells were examined by Western blotting. (J) GAPLINC RNA levels in A549 cells stably expressing control or GAPLINC shRNAs were examined qRT-PCR. (K) GAPLINC RNA levels in A549 cells infected with lentiviruses expressing EV or GAPLINC, were examined by qRT-PCR. Data are presented as means ± SD from three independent experiments, **p < 0.01. (PDF) [file ppat.1011958.s006.pdf]

# S7 Fig

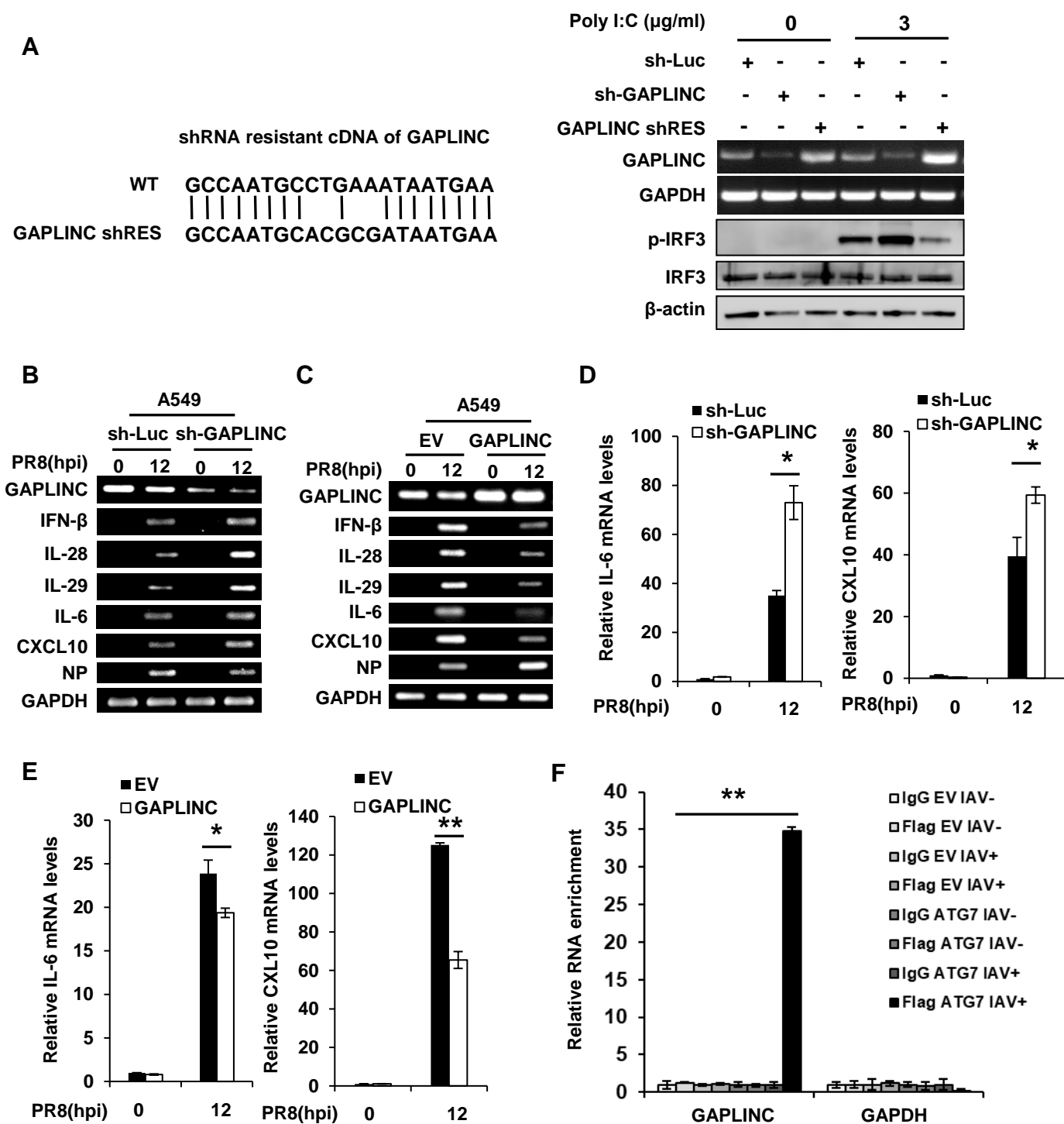

Supplement: S7 Fig — (A) Control and GAPLINC knockdown A549 cells re-expressing an shRNA-resistant form of GAPLINC were transfected with poly(I:C) for 4 h. The levels of p-IRF3 in the cells were examined by Western blotting. (B) RT-PCR analysis of IFN-β, IL-28, IL-29, IL-6 and CXCL10 RNA levels in control and GAPLINC knockdown A549 cells infected with PR8 (MOI = 0.5) for 12 h. (C) The mRNA levels of IFN-β, IL-28, IL-29, IL-6 and CXCL10 in control and GAPLINC overexpressing A549 cells infected with PR8 (MOI = 0.5) for 12 h, were detected by RT-PCR. (D) Control and GAPLINC knockdown A549 cells were infected with PR8 (MOI = 0.5) for 12 h. The RNA levels of IL-6 and CXCL10 were examined by qRT-PCR. (E) qRT-PCR analysis of IL-6 and CXCL10 RNA levels in control and GAPLINC overexpressing A549 cells infected with PR8 (MOI = 0.5) for 12 h. (F) Control and ATG7 overexpressing A549 cells were infected with PR8 (MOI = 0.5) for 16 h, and then subjected to RNA immunoprecipitation (RIP) assays using Flag antibody. GAPDH served as the negative control. RT-PCR data were repeated independently three times with similar results. Shown are representative data of three biologically independent experiments. Data are presented as means ± SD from three independent experiments, *p < 0.05, **p < 0.01. (PDF) [file ppat.1011958.s007.pdf]
