# Supplementary material for: ATG7/GAPLINC/IRF3 axis plays a critical role in regulating pathogenesis of influenza A virus
Source: PLoS Pathog. 2024 Jan 16;20(1):e1011958. doi: 10.1371/journal.ppat.1011958 (PMC10817227; doi:10.1371/journal.ppat.1011958)
Supplement: Attachment — Submitted filename: Responses to Reviewers.doc [file ppat.1011958.s008.doc]

**Responses to Reviewers**

We are very grateful to the reviewers for their critical comments and insightful suggestions. In the new version, we have carefully considered each comment, performed a number of additional experiments to address them and revised the manuscript. The New data are shown in **Figures: 1A, 1C, 1I, 1K, 2F, 6C, and Supplementary Figures: S3C-F, S4A, S5A-F, S6A-K, S7A, S7F**. We are confident that this manuscript has been significantly improved.

**Reviewer's Comments**

**Part I - Summary**

**Reviewer #1:** In this study by Chen et al., the authors investigate the role of autophagy protein ATG7 on influenza A virus (IAV) replication and cell autonomous immune responses. The authors report that shRNA knockdown of ATG7 in the human lung epithelial cell line A549 decreased IAV replication. In contrast, overexpression (OE) of ATG7 in A549 cells increased IAV replication, indicating that ATG7 promotes IAV replication. These findings are further supported by in vivo studies in a tamoxifen inducible Atg7 ko mouse model, where loss of Atg7 decreased IAV viral titer in the lungs. Through RNASeq analysis, the authors identified a non-coding RNA GAPLINC that was lower in IAV infected ATG7 KD cells. shRNA KD or OE of GAPLINC decreased or increased IAV replication, respectively. Interestingly, OE of GAPLINC in ATG7 KD cells was sufficient to rescue IAV replication, indicating that GAPLINC acts downstream of ATG7 and that the lowered IAV replication in ATG7 KD cells is due to decreased GAPLINC levels. Next, the authors investigated if ATG7 and GAPLINC promotes replication by reducing RLR signaling and report that ATG7 suppresses the phosphorylation of IRF3, thereby promoting IAV replication. Similarly, the non-coding RNA GAPLINC reduced the levels of p-IRF3. The authors conclude that ATG7 and GAPLINC promote IAV replication by suppressing IRF3 activation. Although these are interesting findings, the study lacks mechanistic insights into how GAPLINC suppresses IRF3 activation. In addition, the study relies on observations with ATG7 KD/ Atg7 KOs, which needs to be supported by KD or KO of other autophagy genes. Furthermore, some of the RLR suppression studies need to be validated with CRISPR KO or complemented KD cells with shRNA resistant cDNA of ATG7 and GAPLINC.

We are grateful for your consideration and would like to express sincere appreciation for your comments and suggestions. We are pleased that the reviewer thought these are interesting findings. We have seriously considered the comments, performed additional experiments and revised the manuscript accordingly.

To address the molecular mechanisms whereby ATG7 and GAPLINC suppress IRF3 activation, we first examined the association of ATG7 with IRF3. Control and ATG7 overexpressing A549 cells were infected with IAV, and then subjected to immunoprecipitation (IP) assays. As shown in **Fig S5F**, IP of Flag-ATG7 effectively brought down IRF3 in IAV-infected ATG7 overexpressing cells, suggesting the interaction between ATG7 and IRF3 after IAV infection. In addition, we also performed RNA immunoprecipitation (RIP) assays to detect the association of ATG7 with GAPLINC. Control and ATG7 overexpressing A549 cells were infected with IAV, and then subjected to RIP assays. We observed that IP of Flag-ATG7 effectively pulled down GAPLINC in IAV-infected ATG7 overexpressing cells (**Fig S7F**), implying the interaction between ATG7 and GAPLINC upon IAV infection. These data indicate that ATG7 and GAPLINC may form a complex with IRF3 in response to IAV infection, thereby suppressing the activation of IRF3. Further studies are needed to probe the interaction between GAPLINC and IRF3 by RNA pulldown analysis, and to map specific domains responsible for the interaction of ATG7, GAPLINC, and IRF3 in the future. The precise mechanisms whereby ATG7 and GAPLINC inhibit the activation of IRF3 via interaction with IRF3, deserve further investigation in the future. We have discussed this in the Discussion section of revised manuscript.

Moreover, we investigated the function of BECN1 and ATG3, two key proteins involved in autophagy, in IAV infection. A549 cells were transfected with specific siRNAs targeting BECN1 and ATG3 respectively, or control siRNAs, followed by IAV infection. We observed that the mRNA levels of NP were reduced in BECN1 or ATG3 knockdown cells compared with those in control cells after IAV infection (**Fig S6B-C**), indicating that depletion of BECN1 or ATG3 impeded the replication of IAV in A549 cells. Importantly, knockdown of BECN1 or ATG3 had no significant effect on the expression of GAPLINC and activation of IRF3, compared with control cells after IAV infection (**Fig S5B-C, S6B-C**), suggesting that BECN1 and ATG3 might facilitate IAV replication via an autophagy-dependent mechanism that is distinct from the autophagy-independent mechanism by which ATG7 enhances IAV replication via regulating the expression of lncRNA GAPLINC, resulting in the suppression of IRF3 activation and IFN production, thereby restraining host antiviral immunity.

To evaluate the effect of ATG7 and GAPLINC on the activation of IRF3 upon viral infection, as suggested we generated ATG7 knockdown A549 cells re-expressing an shRNA-resistant form of ATG7, followed by transfection with poly(I:C), a dsRNA mimic to activate IRF3 without induction of autophagy (**Fig S5D-E**). It was observed that ATG7 knockdown led to a significant increase in the phosphorylation levels of IRF3 induced by poly(I:C) (**Fig S5E**). Notably, re-introduction of the ATG7 reversed the enhanced IRF3 phosphorylation caused by loss of ATG7 in the knockdown cells following poly(I:C) transfection (**Fig S5E**). In addition, we also generated GAPLINC knockdown A549 cells re-expressing an shRNA-resistant form of GAPLINC, followed by transfection with poly(I:C) (**Fig S7A**). Higher levels of phosphorylated IRF3 were observed in GAPLINC knockdown cells than those in control cells after poly(I:C) transfection, and addback of GAPLINC reversed the enhanced IRF3 phosphorylation caused by GAPLINC deficiency in the knockdown cells following poly(I:C) transfection (**Fig S7A**). These results suggest that ATG7 and GAPLINC suppress IRF3 activation during the viral infection.

**Reviewer #2:** Biao Chen et., al demonstrate ATG7, an essential autophagy effector enzyme, suppresses IRF3 activation and interferon production via lncRNA GAPLINC, revealing an autophagy-independent mechanism whereby ATG7 restrains host innate immunity and unveiling a critical role of ATG7/GAPLINC/IRF3 axis in regulating IAV pathogenesis. These results show that ATG7 has multiple biological roles beyond autophagy, and provide an important insight into the complicated interplay between host and IAV. In general, there are many points involved in the manuscript with large workload and the experimental results are clear and the evidence is relatively sufficient.

We are grateful for the reviewer’s careful analysis of our work and helpful comments. We are pleased that the reviewer thought there are many points involved in the manuscript with large workload and the experimental results are clear and the evidence is relatively sufficient. We have seriously considered each comment and suggestion, completed additional experiments to address them and revised the manuscript.

**Part II – Major Issues**

**Reviewer #1:**

1. The lack of mechanistic insights into how ATG7 and GAPLINC suppress IRF3 activation. The role of autophagy in RLR signaling and GAPLINC in the suppression of inflammatory gene expression has been reported by others. I think further investigation of the underlying mechanisms will strengthen the manuscript.

A: Thanks for the reviewer’s comment. To address the molecular mechanisms whereby ATG7 and GAPLINC suppress IRF3 activation, we first determined whether ATG7 interacts with IRF3. Control and ATG7 overexpressing A549 cells were infected with IAV, and subjected to immunoprecipitation (IP) assays. Indeed, IP of Flag-ATG7 effectively brought down IRF3 in IAV-infected ATG7 overexpressing cells (**Fig S5F**), suggesting the interaction between ATG7 and IRF3 after IAV infection. In addition, we also performed RNA immunoprecipitation (RIP) assays to detect the association of ATG7 with GAPLINC. Control and ATG7 overexpressing A549 cells were infected with IAV, and subjected to RIP assays. We observed that IP of Flag-ATG7 effectively pulled down GAPLINC in IAV-infected cells (**Fig S7F**), implying the interaction between ATG7 and GAPLINC following IAV infection. These data suggest that ATG7 and GAPLINC may form a complex with IRF3 in response to the viral infection, thereby suppressing the activation of IRF3. Further studies are needed to probe the interaction between GAPLINC and IRF3 by RNA pulldown analysis, and to map specific domains responsible for the interaction of ATG7, GAPLINC, and IRF3 in the future. The precise mechanisms whereby ATG7 and GAPLINC inhibit the activation of IRF3 via interaction with IRF3, deserve further investigation in the future. We have discussed this in the Discussion section in the revised manuscript.

2. It is unclear how the expression of GAPLINC is lowered by loss of ATG7.

A: Thanks for the reviewer’s comment. A recent study has revealed that downregulation of GAPLINC in response to inflammation is dependent on NF-κB signaling^[1]^. This prompted us to evaluate the role of NF-κB in regulation of GAPLINC expression by ATG7 during IAV infection. To test this, control and ATG7 knockdown A549 cells were pretreated with DMSO or an NF-κB inhibitor BAY11-7082, followed by IAV infection. In the absence of BAY11-7082 treatment, the expression of GAPLINC was decreased in ATG7 knockdown A549 cells compared with control cells upon IAV infection (**Fig S6G**). In contrast, the downregulation of GAPLINC caused by loss of ATG7 in the cells was impaired compared with control cells after IAV infection when the cells were treated with BAY11-7082 (**Fig S6G**), indicating that ATG7-mediated regulation of GAPLINC is associated with NF-κB signaling. Next, we further examined the phosphorylation of p65, a subunit of NF-κB, in control and ATG7 knockdown A549 cells infected with IAV. Higher levels of phosphorylated p65 were observed in ATG7 knockdown cells than those in control cells after IAV infection (**Fig S6H**), while overexpression of ATG7 led to a significant decrease in the phosphorylation levels of p65 induced by IAV infection (**Fig S6I**). These results suggest that ATG7 may positively regulate the expression of GAPLINC via inhibition of NF-κB activation during IAV infection.

Reference

[1] Apple Cortez Vollmers, Sergio Covarrubias, et al. A conserved long noncoding RNA, GAPLINC, modulates the immune response during endotoxic shock. ***Proc Natl Acad Sci U S A***. 2021 Feb 16;118(7):e2016648118.

3. It needs to be demonstrated if there are changes in ATG7 and GAPLINC during IAV infection and how this impacts the overall IAV life cycle.

A: We agree that it is necessary to determine if there are changes in ATG7 and GAPLINC during IAV infection. Thus, we have examined the expression of GAPLINC and ATG7 during IAV infection. We found that GAPLINC was downregulated in a virus dose- and infection time-dependent manner, while no significant changes in ATG7 protein levels were observed during IAV infection (**Fig S6D-E**). In addition, our data showed that the downregulation of GAPLINC during IAV infection was impaired in A549 cells treated with an NF-κB inhibitor BAY11-7082, relative to non-treated cells after IAV infection (**Fig S6F**). Together, these data imply that GAPLINC expression is negatively regulated by NF-κB signaling, while ATG7 enhances GAPLINC expression via suppression of NF-κB activation during IAV infection.

4. Are the levels of Gaplinc lower in Atg7 KO mouse lungs? The findings will be strengthened if the authors could demonstrate their findings from A549 cells in mouse Atg7 ko cells.

A: We are grateful for the reviewer’s helpful suggestion. We examined the levels of GAPLINC in the lungs of ATG7 conditional knockout or WT mice challenged with IAV infection. Consistent with the data obtained from *in vitro* experiments (**Fig 6A-B**), *in vivo* studies showed that ATG7 conditional knockout mice had a significantly decreased expression of GAPLINC in the lungs compared with the control mice infected with IAV (**Fig 6C**). Together, both *in vitro* and *in vivo* data reveal that ATG7 enhances the expression of GAPLINC during IAV infection.

5. The data for autophagy independent role of ATG7 is weak. The majority of the data relies on chemical inhibitors, which can also block IAV infection by blocking endosomal acidification (HCQ).

A: Thanks for the reviewer’s comment. To further address role of ATG7 in IAV infection, control and ATG7 knockdown A549 cells were transfected with siRNAs targeting BECN1 or ATG3, two key proteins involved in autophagy, followed by IAV infection. We observed that silencing of BECN1 or ATG3 could not block the attenuation of IAV replication caused by loss of ATG7 in the knockdown cells compared with control cells after IAV infection (**Fig S3C-D**). Moreover, we observed that overexpression of ATG7 still promoted the replication of IAV compared with control cells, although BECN1 or ATG3 was depleted in the cells (**Fig S3E-F**), indicating that ATG7 might regulate the replication of IAV, only to some extent, requiring intact autophagy.

Importantly, we constructed two ATG7 mutants devoid of the ability to efficiently regulate autophagy, including the active-site mutant (ATG7^C572S^), and the mutant defective in the formation of the E2-substrate intermediate of ATG3 and LC3 (ATG7^FAPtoDDD^). Then, we generated A549 cells expressing either WT ATG7, each ATG7 mutant, or EV control, followed by infection with IAV (**Fig 3E**). Overexpression of ATG7 WT led to an enhanced replication of IAV compared to EV control (**Fig 3F**). Interestingly, overexpression of ATG7 mutants also significantly increased IAV replication (**Fig 3F**), although the autophagy was suppressed in cells expressing the ATG7 mutants (**Fig 3E**), demonstrating an autophagy-independent pathway utilized by ATG7 to facilitate the IAV replication. In addition, we generated ATG7 knockdown A549 cells re-expressing either WT or the mutants of ATG7, followed by infection with IAV (**Fig 3G**). ATG7 knockdown significantly decreased IAV titers (**Fig 3H**). Notably, addback of either WT or mutants of ATG7 recovered the attenuated IAV replication caused by loss of ATG7 in the cells, despite defected autophagy (**Fig 3H**). Together, these results provide strong evidence that ATG7 promotes the replication of IAV in both autophagy-dependent and -independent manners.

**Reviewer #2**

The logic of the article is a bit confusing and needs to be revised. For example, the authors found that ATG7 could promote IAV infection in an autophagy-independent manner, and then found and explained that lncRNA GAPLINC was downregulated in ATG7 knockdown A549 cells immediately. Later, the authors directly jumped to the context of innate immunity response by ATG7 and guess lncRNA GAPLINC may play critical roles in it. Finally, the relationship between these three was linked together by all the authors’ suppose instead of experimental results. As a result, the research content does not demonstrate a hierarchical and closely connected logic.

Actually, when the authors found that ATG7 could promote IAV infection in an autophagy-independent manner and in order to clarify this molecular mechanism, RNA-Seq experiments were conducted. Compared with RNA-seq results of ATG7 wild-type and knockdown cells, finding that interferon type I and III were upregulated in ATG7 knockdown cells, indicating that ATG7 may regulate IAV infection by influencing innate immune response. Then, experiments were conducted to demonstrate that ATG7 could assist in IAV infection by inhibiting the phosphorylation of IRF3. Next, how does ATG7 regulate this process? Then according to RNA-seq results and it came to the relevant content of lncRNA GAPLINC. This kind of logic is more compact and rigorous.

A: Thanks for the reviewer’s suggestion. In the new version, we have revised the manuscript to make the logic more compact and rigorous.

**Part III – Minor Issues**

**Reviewer #1**

1. Most of the data presented in the manuscript does not indicate the time points for analysis (hpi or dpi?)

A: As suggested, we have added the time points for analysis in the revised manuscript.

2. For hemagglutination titers, the y-axis label should say HA titer and not viral titers.

A: As suggested, we have modified the y-axis label for hemagglutination titers in the revised manuscript.

3. There is a two-fold difference in viral titers. Surprisingly, the NP levels on the western blot are so different.

A: Indeed, sometimes we observed that the viral NP levels on Western blotting are big different although there is a two-fold difference in viral titers. Data shown in Fig 1C are another representative of three biologically independent experiments with similar results.

4. Nomenclature for mouse gene names are incorrect.

A: We have corrected this in the revised manuscript.

5. It will be informative for the readers to show the individual mouse lung titers as a scatter plot instead of a bar graph.

A: We have shown the individual mouse lung titers as a scatter plot in the revised manuscript.

6. Timeline between Tamoxifen injection and viral infection is not clear.

A: Tamoxifen (20 mg/ml, dissolved in a mixture of 98% corn oil and 2% ethanol) was delivered into 7 weeks old ATG7^flox/flox^/UBC-CreERT2 mice by intraperitoneal injection (100 µg/g body weight, once daily for 5 consecutive days). One day after the last tamoxifen treatment, mice were inoculated intranasally with PR8, or were injected intramuscularly with PRV. We have added this in the Materials and Methods section in the revised manuscript.

7. Some of the graphs are overlapping and the legends/numbering are blocked.

A: We have modified this in the revised manuscript.

8. GAPLINC- RNASeq data doesn’t include mock controls. It is important to include this data as prior studies indicate that KD of GAPLINC increases the basal expression levels of inflammatory cytokines.

A: Thanks for the reviewer’s suggestion. We have included mock controls for RNA-Seq data in the revised manuscript (Fig S4A and S6A).

**Reviewer #2:**

1. S1A Fig showed that A549 cells expressing ATG7 shRNAs could still detect ATG7 protein, but in Fig 3G or S3B Fig, A549 cells expressing ATG7 shRNAs seemed appeared no ATG7, just like ATG7 knockout cells. It needs to be modified and explained.

A: Thanks for the reviewer’s comment. Indeed, sometimes we observed that the efficiency of ATG7 knockdown in A549 cells was very high. Data shown in Fig 3G and S3B are representative of three biologically independent experiments with similar results.

2. In Fig 2A, authors detected ATG7 protein levels in ATG7 flox/flox/UBC-CreERT2 mice tissues, but instead of thymus, all the tissues showed a high level of ATG7 in ATG7-/- mice. I don't know whether “ATG7 knockout mice” described in the article are suitable.

A: We have replaced “ATG7 knockout mice” with “ATG7 conditional knockout mice” in the revised manuscript.

3. In the context of effects of ATG7 on the IRF3 activation, authors only checked IRF3 phosphorylation. What’s the effects of ATG7 on IRF3 dimerization and nuclear translocation. Authors should add these results to make the article conclusion stronger.

A: Thanks for the reviewer’s suggestion. To evaluate the effect of ATG7 on IRF3 nuclear translocation, control and ATG7 knockdown A549 cells were infected with SeV, and subjected to immunofluorescence (IF) assays. We observed that the nuclear translocation of IRF3 was enhanced in ATG7 knockdown A549 cells compared with control cells following SeV infection (**Fig S5A**), suggesting that ATG7 inhibited the activation of IRF3 during viral infection.
